# The Pacifier

**Published:** 1914-04

**Authors:** Marianna Wheeler

**Affiliations:** Superintendent for Fifteen Years of the Babies’ Hospital in New York


### The Pacifier.

BY MARIANNA WHEELER, SUPERINTENDENT FOR FIFTEEN YEARS OF THE BABIES' HOSPITAL IN NEW YORK.

No matter where one is, whether in the crowded city, the isolated country districts or remote foreign countries, one is certain to see a baby with a pacifier, or as they are often called, a silencer, in its mouth. I wonder if the mothers who give these little abominations to their infants realize what a menace they are to the health of their children. Almost, without exception, they cause harm and suffering to the children upon whom they are inflicted. I have never known of an infant having derived the slightest benefit from using one, but do know of hundreds of cases where they have been the cause of suffering to the child. When a mother is asked why she gives her baby one of those things, her reply is invariably, "to keep the baby quiet," or to stop his crying. This is really a very poor and thoughtless excuse. It would be much more sensible for the mother to make some effort to learn the cause of the baby's crying and eliminate that cause if possible. An infant may cry for several reasons. It affords a very young baby much pleasure to cry hard enough to expand his lungs; this is also quite necessary to a healthy infant, and it seems heartless to deprive him of this harmless pleasure. He is also just as likely sometimes to cry from a fit of temper. As we are all liable to have moderate attacks of temper occasionally, the baby ought to be entitled to a little lapse in this respect once in a while. Surely, indulgence, in the shape of a pacifier, will not help a child overcome temper or acquire self-control. Babies cry from pain and from hunger, but does any mother expect a pacifier to take the place of food or to soothe and stop pain? It is not only imposing upon the innocent babe, but doing a decided wrong to give him one of these things or any other similar device. They are most unhealthy, for their is no surer means of carrying germs of disease. Who has not seen one of these little germ carriers tied to a baby's wrist or the side of his carriage and dangling down against the wheels, as the child is trundled through the city streets, or dusty or muddy roads of the small town? They are dropped on the sidewalk or road, on the floors of the house, store or dirty car. When picked up, they are usually given a careless wipe on apron, dress, or anything else that is handy, even to a soiled handkerchief, and then tucked back in the infant's mouth. But this is not by any means all the harm they are guilty of inflicting. The constant effort made in sucking on one of these devices causes the soft tissue at base of the nostrils to enlarge and form a spongy growth which blocks the nostrils, making it impossible for the child to breathe through the nose. This growth is known by the term "adenoids" and the only remedy for these is to have them removed by a surgical operation. If these are allowed to remain, it means a lifetime of nose and throat torture, and usually deafness. While this growth remains to block up the nostrils, children become dull and mentally apathetic. Normal intellectual conditions, however, return when the growth is removed. A celebrated throat specialist states that two-thirds of the cases of adenoids that come to him for treatment are caused by the children having had a pacifier or being allowed to suck their thumbs in infancy.

